# METTL14 Regulates Myocardial Infarction Progression via m^6^A‐Dependent Modulation of OTUD1‐Mediated Deubiquitination of DUSP6

**DOI:** 10.1002/kjm2.70193

**Published:** 2026-03-30

**Authors:** Cheng‐Cheng Wei, Ya‐Fang Shen, Jin‐Yu Zhang

**Affiliations:** ^1^ Hangzhou Medical College Department of Cardiology, Tongxiang First People's Hospital of Zhejiang Province Tongxiang China; ^2^ Zhejiang Chinese Medical University Teaching Base Outpatient Clinic, Tongxiang City Traditional Chinese Medicine Hospital Tongxiang China

**Keywords:** DUSP6, m^6^A modification, METTL14, myocardial infarction, OTUD1

## Abstract

Myocardial infarction (MI) is the most severe clinical manifestation of coronary artery diseases (CVD) and serves as a critical driver of sudden cardiac death and heart failure (HF). Its pathophysiology begins with the abrupt cessation of coronary blood flow, leading to severe ischemia and subsequent cardiomyocyte necrosis. This study aimed to investigate the molecular mechanisms by which METTL14 regulates the progression of MI in mice via the OTUD1/DUSP6 signaling axis. An MI mouse model was established by ligating the left anterior descending (LAD) coronary artery. The progression of MI was evaluated through echocardiography, HE staining, Masson's trichrome staining, TUNEL assay, and assessment of inflammatory cytokines. Mechanistically, Me‐RIP, PAR‐CLIP Co‐IP, and protein stability assays were performed to dissect the interactions within the METTL14/OTUD1/DUSP6 axis. Our results demonstrated that METTL14 was highly expressed in the MI mouse model. Silencing METTL14 significantly reduced the left Ventricular Internal Diameter at end‐diastole (LVIDd) and left Ventricular Internal Diameter at end‐systole (LVIDs), increased ejection fraction (EF) and fractional shortening (FS), and attenuated histopathological damage, apoptosis, and the levels of inflammatory cytokines (TNF‐α and IL‐β). Further analysis revealed that METTL14 promotes OTUD1 mRNA stability and expression by modulating its m^6^A modification. In turn, METTL14 influences DUSP6 expression by regulating OTUD1‐mediated ubiquitination. Collectively, silencing METTL14 modulates the MI disease process through the OTUD1/DUSP6 signaling axis, suggesting that METTL14 plays a pivotal role in MI progression. These findings indicate that targeting METTL14 may represent a potential therapeutic strategy to alleviate pathological injury, apoptosis, and inflammation during MI.

## 1 Introduction

Myocardial infarction (MI) remains a predominant contributor to global morbidity and mortality, representing the most severe clinical manifestation of coronary artery disease (CAD) and a key driver of sudden cardiac death and heart failure (HF) [1]. MI results from an abrupt interruption of coronary blood flow, which gives rise to severe ischemia and subsequent necrosis of cardiomyocytes within the affected myocardial region [2]. Although the implementation of timely reperfusion, advanced antiplatelet regimens, and standardized acute‐phase management has markedly improved short‐term survival, many patients develop adverse cardiac remodeling and progressive HF during the post‐infarction period [3]. This highlights the urgent need for novel therapeutic strategies that can inhibit cardiomyocyte death, reduce inflammation, and promote myocardial repair.

Epigenetic regulation, particularly N6‐methyladenosine (m^6^A) RNA modification, has emerged as a crucial mechanism in cardiovascular diseases, including MI [4]. m^6^A is installed by a methyltransferase complex in which METTL3 and METTL14 are the primary components, with METTL14 acting as an essential catalytic subunit [5]. In addition, METTL14 serves as a critical regulator of cardiomyocyte survival; its inhibition effectively mitigates cell death by suppressing mitochondrial fragmentation and preventing myofibrillar transformation [6]. Recent studies have found that METTL14‐dependent m^6^A modification influences cardiomyocyte pyroptosis and inflammatory responses during MI, highlighting its potential as a therapeutic target [7, 8]. In addition to modulating cell death pathways, m^6^A modification also controls the expression and stability of key downstream transcripts involved in pathological remodeling [9, 10]. Nevertheless, the specific downstream targets and signaling pathways modulated by METTL14 under myocardial injury conditions have yet to be fully elucidated.

OTU domain‐containing protein 1 (OTUD1) is a deubiquitinating enzyme (DUB) implicated in the regulation of inflammatory signaling and protein stability. Functionally, OTUD1 suppresses inflammation by removing K63‐linked ubiquitin chains from substrates, such as RIPK1 and IRF3 [11]. In the cardiovascular context, OTUD1 expression is upregulated in response to pathological stimuli [12, 13], where it promotes cardiac hypertrophy and fibrosis via the activation of signaling cascades, including STAT3 and the ASK1‐p38/JNK axis [14]. Previous studies have demonstrated the post‐transcriptional and post‐translational epigenetic regulation of OTUD1 in CADs. For instance, OTUD1 promotes HF by deubiquitinating and stabilizing PDE5A, thereby dysregulating cGMP‐PKG‐SERCA2a signaling and calcium homeostasis in cardiomyocytes [15], as well as promoting ischemia/reperfusion‐induced heart injury by deubiquitinating RACK1 [16]. Intriguingly, recent evidence suggests that METTL3‐mediated m^6^A modification enhances the stability of OTUD1 mRNA, indicating that OTUD1 may act as a downstream effector of m^6^A signaling in cardiac pathology [14]. However, whether METTL14‐mediated m^6^A similarly governs OTUD1 expression and activity during MI remains unknown.

As a ubiquitin‐sensitive signaling node, OTUD1 has the potential to modulate downstream phosphatases, such as dual‐specificity phosphatase 6 (DUSP6). Also known as MKP3, DUSP6 is a cytoplasmic phosphatase that selectively dephosphorylates ERK, thereby regulating the MAPK cascade [17]. Loss of DUSP6 has been linked to improved post‐MI recovery, largely through the suppression of neutrophil‐driven myocardial injury [18]. DUSP6 serves as a pro‐inflammatory mediator in the post‐MI microenvironment, with its activation promoting the differentiation of bone marrow cells into macrophages, thereby elevating the levels of various cytokines and chemokines [19]. However, DUSP6 has also been reported to serve as a pivotal downstream effector of TRIM11, playing a cardioprotective role by inhibiting ferroptosis and mitigating mitochondrial damage during acute MI [20]. DUSP6 levels are stringently controlled by ubiquitination and subsequent proteasomal degradation, with multiple DUBs modulating its stability in various disease settings [21]. These observations raise the possibility that OTUD1 may influence post‐infarction cardiac remodeling by regulating DUSP6; however, direct evidence for an OTUD1‐DUSP6 regulatory axis in cardiomyocytes under MI conditions is currently lacking. Taken together, we hypothesize that METTL14 may promote MI progression through the m^6^A‐mediated upregulation of OTUD1, which in turn stabilizes DUSP6 by deubiquitination, thereby contributing to mitochondrial dysfunction, apoptosis, and adverse cardiac remodeling. Deciphering this METTL14‐m^6^A‐OTUD1‐DUSP6 axis may uncover new molecular targets for cardioprotective therapy following MI.

## 2 Materials and Methods

### 2.1 In Silico Analysis

Potential m^6^A methylation sites within the nucleotide sequence of OTUD1 were predicted using the SRAMP database (http://www.cuilab.cn/). Ubiquitination modification sites in the amino acid sequence of DUSP6 were predicted using the PhosphoSite database (https://www.phosphosite.org/).

### 2.2 Establishment of the Mouse MI Model

C57BL/6 mice aged 7‐weeks‐old were obtained from Hunan SJA Laboratory Animal Co. Ltd. (Changsha, China). All animals were housed in a specific pathogen‐free (SPF) facility under standard conditions (22°C ± 3°C, 60% ± 5% humidity, 12‐h light/dark cycle) and provided food and water ad libitum. After a one‐week acclimation period, MI was induced by ligating the left anterior descending (LAD) coronary artery. Mice were anesthetized with isoflurane (3% for induction, 2% for maintenance). Following shaving and disinfection of the thoracic region, a suture was placed around the upper incisors to extend the neck. The tongue was gently retracted with forceps, and a 20‐G catheter was inserted into the trachea and connected to a rodent ventilator through a Y‐shaped adaptor. Mice (~25 g) were mechanically ventilated with 100% oxygen at a respiratory rate of 130 breath/min and a tidal volume of 225 μL. The heart was exposed by performing a left thoracotomy at the third to fourth intercostal space adjacent to the sternum. A small retractor was applied to improve visualization, after which the pericardium was carefully incised. The LAD coronary artery was then ligated with an 8–0 silk suture (Ethicon). Slight over‐inflation of the lungs was performed to evacuate air from the pleural cavity, after which the intercostal space and overlying skin were sutured with 6–0 silk sutures (Ethicon) [22]. For the sham group (*n* = 5), all surgical procedures were performed without LAD ligation. The MI groups included: MI, MI + sh‐NC, MI + sh‐METTL14, MI + sh‐METTL14 + oe‐NC, MI + sh‐METTL14 + oe‐OTUD1, and MI + sh‐METTL14 + oe‐DUSP6 (*n* = 5 per group). At 24 h post‐MI modeling, mice with successful MI were administered an intracardiac injection of lentivirus vectors (5 μL, 10^7^ TU/mL) carrying either shRNA or overexpression constructs targeting METTL14, OTUD1, or DUSP6 [22]. All animal procedures followed the Guide for the Care and Use of Laboratory Animals and were approved by Animal Care and Use Committee (IACUC) of Tongxiang First People's Hospital of Zhejiang Province. Surgeries were carried out under anesthesia, with every effort taken to minimize suffering. Mice were humanely euthanized at the completion of the study.

### 2.3 Echocardiographic Assessment

After anesthesia, the mice were positioned supine and secured onto the imaging platform. Left ventricular end‐diastolic diameter (LVIDd), fractional shortening (FS), left ventricular end‐systolic diameter (LVIDs), and ejection fraction (EF) were continuously recorded over three cardiac cycles. Cardiac function was evaluated by analyzing these parameters [23, 24].

### 2.4 Hematoxylin and Eosin (HE) Staining

Paraffin‐embedded myocardial tissue sections from each group were baked in a 60°C oven for 30 min. The sections were first treated with xylene to remove paraffin, then passed through a graded ethanol series for rehydration and finally washed with deionized water. Hematoxylin staining was performed for 3–5 min, followed by rinsing in deionized water. The sections were then differentiated in 1% hydrochloric acid‐alcohol for 20 s, blued in 1% ammonia water for 30 s, and rinsed again with deionized water. Counterstaining was performed with 1% eosin solution for 5 min, followed by rinsing in tap water for 5 min and then in deionized water for 1 min. For dehydration and clearing, the sections were sequentially immersed in 75% ethanol (5 min), 90% ethanol (5 min), 95% ethanol (5 min), and absolute ethanol (5 min), followed by xylene twice for 10 min. After drying, coverslips were mounted. The sections were imaged under a light microscope.

### 2.5 Masson's Trichrome Staining

Myocardial specimens were fixed in 4% paraformaldehyde, embedded in paraffin, and sectioned. The sections were dehydrated using a graded ethanol series and stained using a Masson's trichrome staining kit (KeyGen Biotech, China) as recommended by the manufacturer. A light microscope (BX43; Olympus, Tokyo, Japan) was used to acquire images of the stained sections. The collagen volume fraction was determined by dividing the collagen‐positive area by the total tissue area.

### 2.6 TUNEL Staining

Paraffin‐embedded myocardial tissue sections (4‐μm‐thick) were dewaxed and rehydrated using standard protocols, followed by rinsing with phosphate‐buffered saline (PBS). The slides were incubated with Proteinase K working solution at 37°C for 20 min. TUNEL staining was then performed by covering the sections with TdT enzyme diluted at a ratio of 30% in reaction buffer (ApopTag Peroxidase In Situ Apoptosis Detection Kit; Chemicon, USA). Incubation was carried out in the dark at 37°C for 60 min, and staining was subsequently developed with DAB.

### 2.7 Enzyme‐Linked Immunosorbent Assay (ELISA)

Mouse blood samples were centrifuged to obtain serum, and the concentrations of TNF‐α (PT512; Beyotime, China) and IL‐1β (PI301; Beyotime) were determined with ELISA kits.

### 2.8 Western Blot

Tissue samples were digested with trypsin and lysed using enhanced RIPA lysis buffer supplemented with protease inhibitors (Boster, Wuhan, China). Protein levels were assessed by the BCA method (Boster). Equivalent protein samples were resolved by SDS‐PAGE and then transferred to PVDF membranes at a constant voltage of 80 V. After blocking with blocking buffer for 1 h at room temperature, the membranes were maintained at 4°C overnight with the following antibodies: anti‐METTL14 (ab309096, 1:1000; Abcam, UK), anti‐OTUD1 (PA5‐107207, 1:5000; Thermo Fisher Scientific, USA), anti‐DUSP6 (ab76310, 1:500; Abcam), and anti‐GAPDH (ab9485, 1:2500; Abcam). Subsequently, the membranes were incubated with HRP‐conjugated goat anti‐rabbit IgG secondary antibody (ab205718, 1:10000; Abcam) at 37°C for 1 h with gentle shaking. After three washes with PBS (5 min each), the protein bands were visualized using enhanced chemiluminescence (ECL) detection reagent and imaged using the SmartView Pro 2000 imaging system (UVCI‐2100; Major Science, USA). The intensity of protein bands was analyzed with Quantity One software (Quantity One 1‐D; Bio‐Rad, Shanghai, China).

### 2.9 Primary Cardiomyocyte Culture

Myocardial tissue was digested in a solution containing 0.4 mg/mL collagenase and 0.6 mg/mL trypsin, prepared in 116 mM NaCl, 20 mM HEPES (pH 7.35), 0.8 mM NaH_2_PO_4_, 5.6 mM glucose, 5.4 mM KCl, and 0.8 mM MgSO_4_. The cells were harvested after centrifugation (60 × *g*, 5 min) and resuspension in medium consisting of 70% DMEM, 15% M199, 15% fetal calf serum (FCS), and 100 U/mL penicillin–streptomycin. The cells were seeded in 60‐mm Primaria culture dishes pre‐coated with 1% gelatin (37°C, 30 min) to remove non‐cardiomyocytes. The non‐adherent cardiomyocytes were then seeded at a density of 1 × 10^6^ cells per 60‐mm gelatin‐coated Primaria dish and incubated at 1°C. After 18 h, cardiomyocytes were rinsed and cultured in serum‐free medium composed of 80% DMEM, 20% M199, and 100 U/mL penicillin–streptomycin. Identification of cardiomyocytes and cell purity measurement was performed via cTnI immunofluorescence: After 3 days of culture, the medium was discarded, and cardiomyocytes were fixed with 4% paraformaldehyde for 10–20 min, followed by overnight incubation with a cTnI polyclonal primary antibody at 4°C. The cells were then incubated with an SABC‐FITC‐conjugated secondary antibody for 2 h in the dark; after DAPI staining for 5 min protected from light, the samples were mounted with an aqueous mounting medium and then observed and photographed under a fluorescence upright microscope. cTnI labels the cytoplasm of cardiomyocytes green; the number of fluorescence‐positive cells was taken as the cardiomyocyte count to calculate the cell purity. A purity of 85% or higher was required for the primary cardiomyocytes.

Once the cells reached ~80% confluence after 24 h of routine culture, Lipofectamine 2000 (Invitrogen) was employed for transfection as recommended by the manufacturer. Cells were divided into the following experimental groups: sh‐NC (transfected with shRNA negative control), sh‐METTL14 (transfected with shRNA targeting METTL14), oe‐NC (transfected with overexpression negative control), oe‐OTUD1 (transfected with overexpression vector for OTUD1), and oe‐METTL14 (transfected with overexpression vector for METTL14). All plasmids for knockdown and overexpression were provided by GenePharma Co. Ltd. (Shanghai, China) [25, 26].

### 2.10 Methylated RNA Immunoprecipitation (Me‐RIP)

Total RNA was extracted from myocardial tissue with the TRIzol reagent, and mRNA was further purified using the PolyATtract mRNA Isolation System (A‐Z5300; Aidetek Biotechnology Co. Ltd., Beijing, China). For the Me‐RIP assay, samples were divided into experimental groups. mRNA was incubated with anti‐m^6^A antibody (ab151230, 1:500; Abcam, Cambridge, UK) or control IgG antibody (ab109489, 1:100; Abcam) that had been pre‐conjugated to Protein A/G magnetic beads in IP buffer (20 mM Tris, pH 7.5; 140 mM NaCl; 1% NP‐40; 2 mM EDTA) for 1 h. The mixture was then incubated overnight at 4°C in IP buffer supplemented with RNase inhibitors and protease inhibitors. After incubation, the beads were washed, and the bound RNA was eluted, then further purified using phenol–chloroform extraction. The enrichment of OTUD1 mRNA was then analyzed using reverse transcription quantitative reverse transcription PCR (RT‐qPCR).

### 2.11 Photoactivatable Ribonucleoside‐Enhanced Crosslinking and Immunoprecipitation (PAR‐CLIP)

Relevant cell suspensions were reacted with 200 μM 4‐thiouridine (4SU) (Sigma‐Aldrich) for 14 h, followed by UV crosslinking at 365 nm with an energy dose of 0.4 J/cm^2^. After cell lysis, IP was performed using anti‐METTL14 antibody at 4°C overnight. [γ‐32P]‐ATP was used to label the precipitated RNA, which was then visualized by autoradiography. For qRT‐PCR analysis, the precipitates were first treated with proteinase K to remove proteins, and RNA was subsequently extracted using TRIzol reagent. The expression level of OTUD1 was then measured using RT‐qPCR.

### 2.12 Co‐Immunoprecipitation (Co‐IP)

Cells were lysed on ice using IP lysis buffer supplemented with protease inhibitors (Chemical Book, Wuxi, China). For each sample, 1 mg of total protein was diluted to the same volume with IP lysis buffer and rotated overnight at 4°C with monoclonal antibodies against OTUD1 or DUSP6. The following day, 20 μL of Protein A + G magnetic beads were added to each sample and incubated for an additional 2 h at 4°C. The beads were then washed five times with IP lysis buffer to remove nonspecific proteins (centrifugation at 2500 rpm for 5 min at 4°C). After the final wash step, the supernatant was removed, and 20 μL of 2× loading buffer was added per tube. The immunoprecipitated proteins were subsequently analyzed by SDS‐PAGE and western blotting [27].

### 2.13 Protein Stability Assay

To evaluate the DUSP6 protein stability, cells were reacted with RIPA buffer (P0013B; Beyotime, China), followed by centrifugation at 12000 r/min to collect supernatant. Prior to analysis, cells were treated with cycloheximide (CHX), a protein synthesis inhibitor, and incubated for the designated time intervals. The proteasome inhibitor MG132 and CHX were purchased from Sigma (USA) and used at working concentrations of 100 μg/mL for CHX and 20 μM for MG132. After treatment, cells were treated with RIPA buffer containing 0.1% SDS, and protein levels were measured using western blotting. Densitometric analysis was performed using ImageJ software [27].

### 2.14 Statistical Analysis

Statistical analyses were performed using SPSS software version 24.0 (IBM SPSS Statistics, Chicago, IL, USA). All data were subjected to tests for normality and homogeneity of variance. Data conforming to a normal distribution were presented as the mean ± standard deviation (SD). Between‐group comparisons were conducted using unpaired Student's *t*‐tests, while intergroup comparisons were performed using one‐way analysis of variance (ANOVA) and Tukey's post hoc test. *p* < 0.05 was considered statistically significant.

## 3 Results

### 3.1 Elevated METTL14 Expression in MI Mouse Models

**FIGURE 1:**
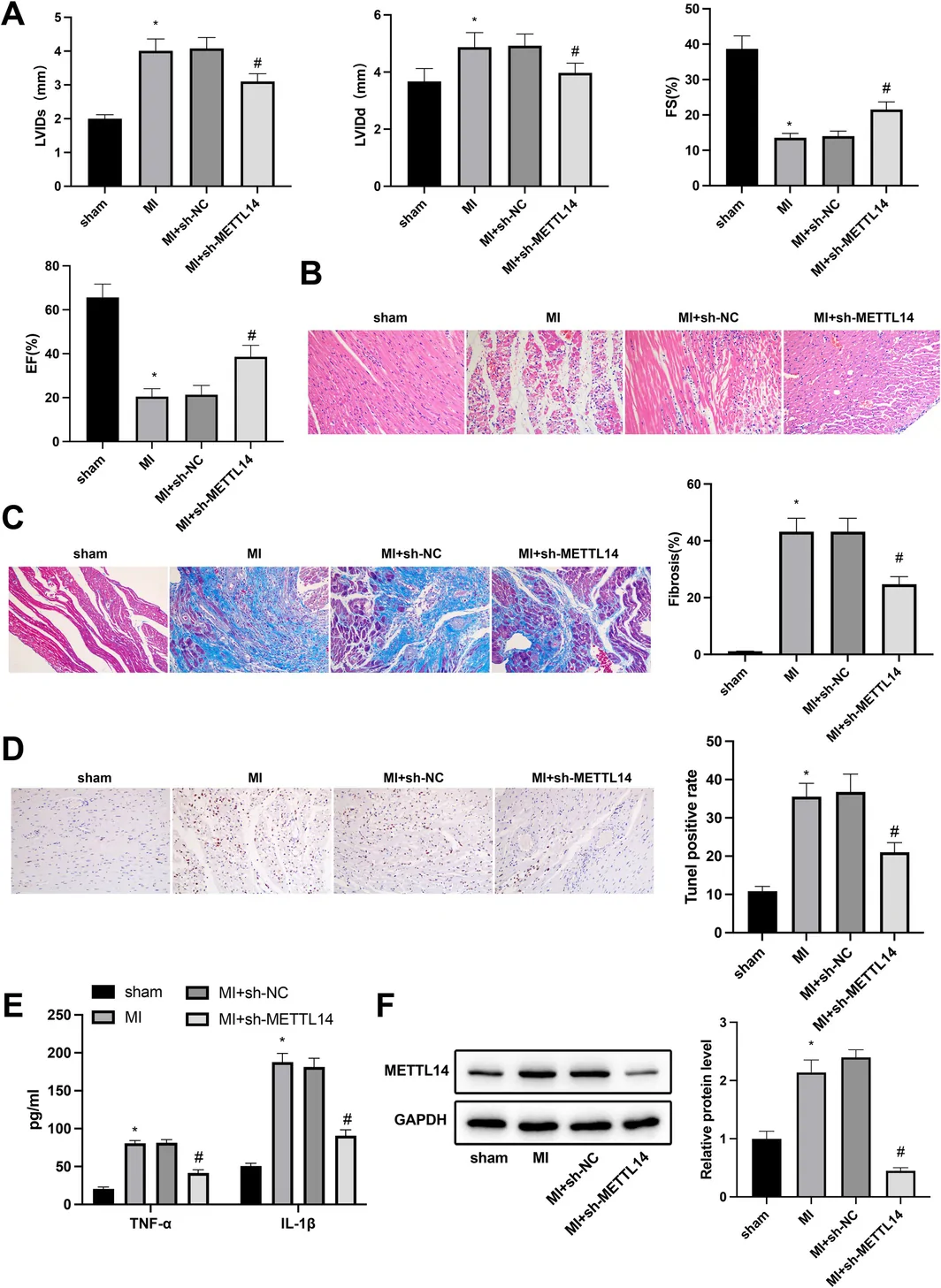
METTL14 is upregulated in a mouse model of MI, and METTL14 silencing attenuates disease progression. (A) Echocardiographic assessment of cardiac function parameters in mice. (B) HE staining of myocardial tissue to evaluate histopathological changes. (C) Masson's trichrome staining to assess myocardial fibrosis. (D) TUNEL staining to detect cardiomyocyte apoptosis. (E) ELISA analysis of serum TNF‐α and IL‐1β levels. (F) Western blot analysis of METTL14 protein expression. Data are presented as mean ± standard deviation (SD). Comparisons among multiple groups were performed using one‐way ANOVA followed by Tukey's post hoc test. *p < 0.05 vs. sham group; #*p* < 0.05 vs. MI + sh‐NC group; *n* = 5.

To explore the role of METTL14 in MI, we established a mouse MI model by permanent ligation of the LAD coronary artery. Echocardiographic analysis revealed that MI mice exhibited significantly increased LVIDd and LVIDs, along with decreased EF and FS compared to the sham group (Figure 1A). Histological examination showed aggravated myocardial damage and increased inflammatory cell infiltration in the MI group, as evidenced by HE staining (Figure 1B), as well as significantly enhanced myocardial fibrosis indicated by Masson's trichrome staining (Figure 1C). TUNEL staining revealed increased cardiomyocyte apoptosis in the infarct border zone of MI mice (Figure 1D). Furthermore, the ELISA results demonstrated elevated TNF‐α and IL‐1β serum levels in the MI group compared to the sham group (Figure 1E). These findings confirm the successful establishment of the MI model.

Western blot analysis revealed that METTL14 expression was significantly elevated in the MI group compared to the sham group (Figure 1F), indicating that METTL14 is upregulated in the MI model. To further investigate the functional role of METTL14 in MI, we silenced METTL14 expression following MI induction. Western blotting confirmed successful knockdown of METTL14, and subsequent analyses were performed to assess cardiac function and injury. Echocardiographic measurements showed that, compared to the MI + sh‐NC group, the MI + sh‐METTL14 group exhibited significantly reduced LVIDd and LVIDs, along with improved EF and FS (Figure 1A). HE staining revealed attenuated myocardial pathological damage and reduced inflammatory cell infiltration in the MI + sh‐METTL14 group (Figure 1B), while Masson staining indicated a significant reduction in myocardial fibrosis (Figure 1C). TUNEL staining showed decreased cardiomyocyte apoptosis in the infarct border zone (Figure 1D), and the ELISA results demonstrated markedly lower serum levels of TNF‐α and IL‐1β (Figure 1E). These findings suggest that METTL14 knockdown mitigates myocardial injury and inflammatory responses, thereby attenuating the progression of MI in mice.

### 3.2 METTL14 Promoted OTUD1 mRNA Stability via Regulating OTUD1 m^6^A Modification

**FIGURE 2:**
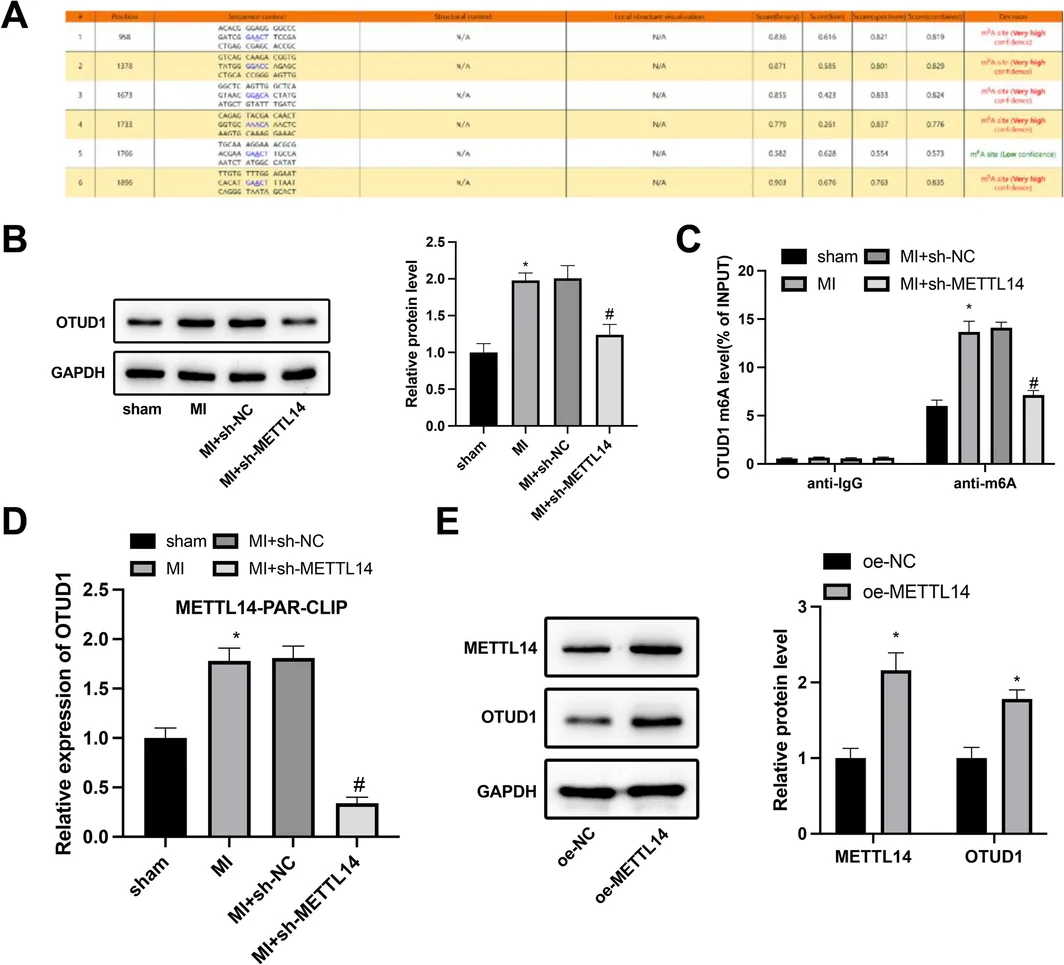
METTL14 promotes OTUD1 expression by regulating its m^6^A modification. (A) Predicted m^6^A modification sites within the OTUD1 sequence based on the SRAMP database. (B) Western blot analysis of OTUD1 protein levels in myocardial tissue. (C) Me‐RIP assay detecting m^6^A modification levels of OTUD1 mRNA in each group. (D) PAR‐CLIP assay assessing the binding interaction between METTL14 and OTUD1 mRNA. (E) Western blot analysis of METTL14 and OTUD1 expression in primary cardiomyocytes. Data are presented as mean ± standard deviation (SD). Comparisons between two groups were performed using unpaired Student's *t*‐test, and multiple group comparisons were analyzed by one‐way ANOVA followed by Tukey's post hoc test. *p < 0.05 vs. sham group; #*p* < 0.05 vs. MI + sh‐NC group; *n* = 5.

Previous studies have reported that OTUD1 deficiency alleviates myocardial hypertrophy and cardiac dysfunction following MI [15]. Bioinformatic analysis using the SRAMP database (http://www.cuilab.cn/) predicted multiple m^6^A modification sites within the OTUD1 sequence (Figure 2A). To determine whether METTL14 regulates OTUD1 expression via m^6^A modification, western blot analysis was performed. OTUD1 expression was found to be significantly elevated in the MI group compared to the sham group but was markedly reduced following METTL14 knockdown (Figure 2B). Me‐RIP assays showed increased m^6^A modification of OTUD1 mRNA in the MI group, which decreased significantly after METTL14 silencing (Figure 2C). PAR‐CLIP assays further confirmed enhanced METTL14 binding to OTUD1 mRNA in MI hearts, which was diminished upon METTL14 knockdown (Figure 2D). Additionally, METTL14 overexpression in primary cardiomyocytes upregulated both the METTL14 and OTUD1 protein levels (Figure 2E). These results indicate that METTL14 promotes OTUD1 expression by enhancing its m^6^A modification and mRNA stability.

### 3.3 METTL14 Influences the Ubiquitination and Expression of DUSP6 by Regulating OTUD1 Expression

**FIGURE 3:**
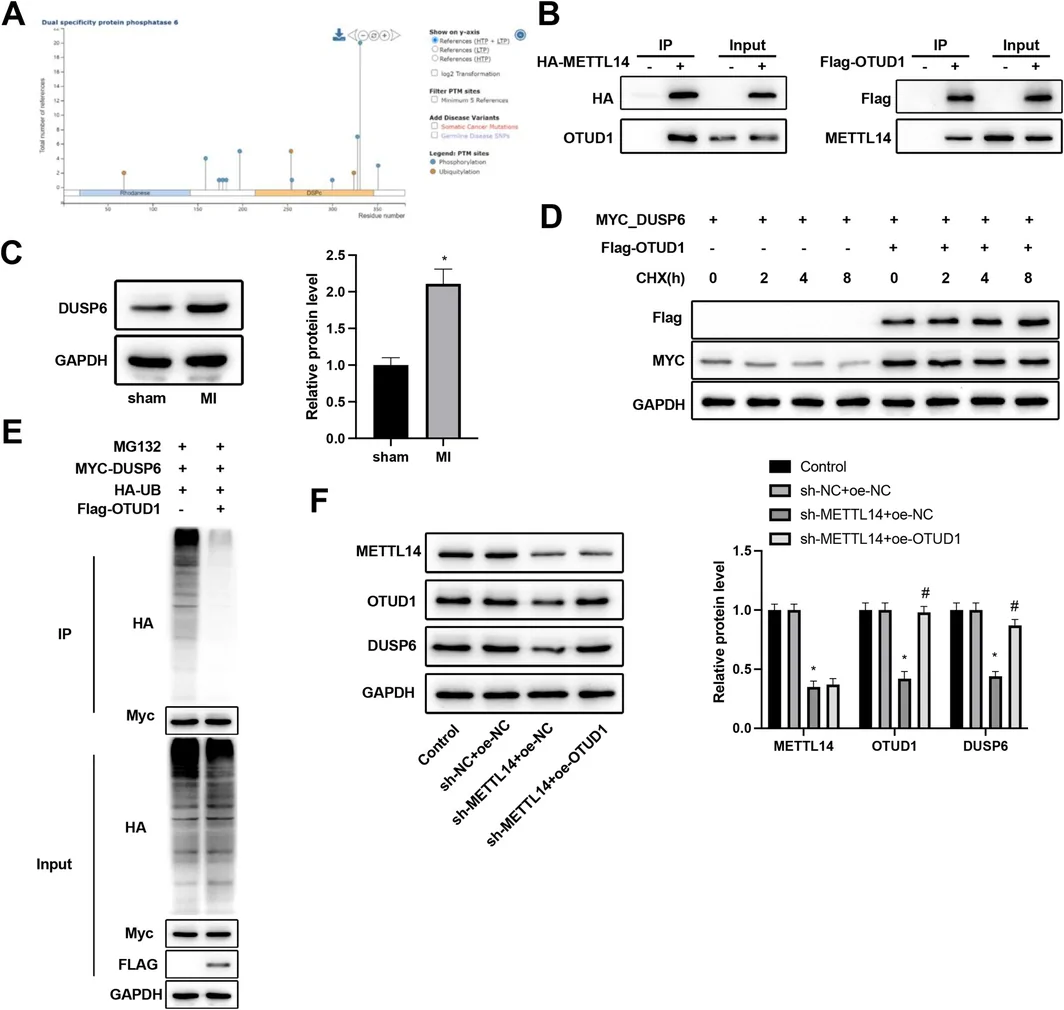
METTL14 regulates DUSP6 expression by modulating OTUD1‐mediated deubiquitination. (A) Predicted ubiquitination sites in DUSP6 based on the PhosphoSite database (https://www.phosphosite.org/). (B) Co‐IP analysis of the interaction between OTUD1 and DUSP6. (C) Western blot analysis of DUSP6 expression levels in each group. (D) Western blot detection of DUSP6 ubiquitination levels. (E) Assessment of DUSP6 protein stability following proteasome inhibition. (F) Western blot analysis of METTL14, OTUD1, and DUSP6 expression in cardiomyocytes. Data are presented as mean ± standard deviation (SD). Comparisons between two groups were conducted using unpaired Student's *t*‐test, and comparisons among multiple groups were analyzed using one‐way ANOVA followed by Tukey's post hoc test. *p < 0.05 vs. sham group or sh‐NC + oe‐NC group; #*p* < 0.05 vs. sh‐METTL14 + oe‐NC group; *n* = 5.

Previous studies have demonstrated that OTUD1 suppresses proteasomal degradation of downstream proteins via deubiquitination [28]. Bioinformatic analysis using the PhosphoSite database (https://www.phosphosite.org/) revealed multiple predicted ubiquitination sites within the amino acid sequence of DUSP6 (Figure 3A). To investigate whether OTUD1 regulates DUSP6 expression through ubiquitination in MI, we first performed Co‐IP assays and confirmed the interaction between OTUD1 and DUSP6 in myocardial tissue (Figure 3B). Western blot analysis showed that DUSP6 expression was significantly upregulated in the MI group compared to the sham group (Figure 3C). Following CHX treatment, both the OTUD1 and DUSP6 expression levels decreased, indicating that CHX suppresses OTUD1‐mediated deubiquitination and affects DUSP6 stability (Figure 3D). Furthermore, MG132 treatment following OTUD1 knockdown decreased DUSP6 protein stability, while OTUD1 overexpression attenuated DUSP6 ubiquitination (Figure 3E). To determine whether METTL14 regulates DUSP6 expression by modulating OTUD1, we examined the expression of METTL14, OTUD1, and DUSP6 in primary cardiomyocytes. Compared with the sh‐NC + oe‐NC group, the expression of METTL14, OTUD1, and DUSP6 was markedly decreased in the sh‐METTL14 + oe‐NC group, while the OTUD1 and DUSP6 expression levels were restored upon OTUD1 overexpression in the sh‐METTL14 + oe‐OTUD1 group (Figure 3F). Collectively, these results suggest that METTL14 regulates DUSP6 expression by modulating OTUD1‐mediated deubiquitination.

### 3.4 Knockdown of METTL14 Regulated MI Progression in Mice by Mediating the OTUD1/DUSP6 Axis

**FIGURE 4:**
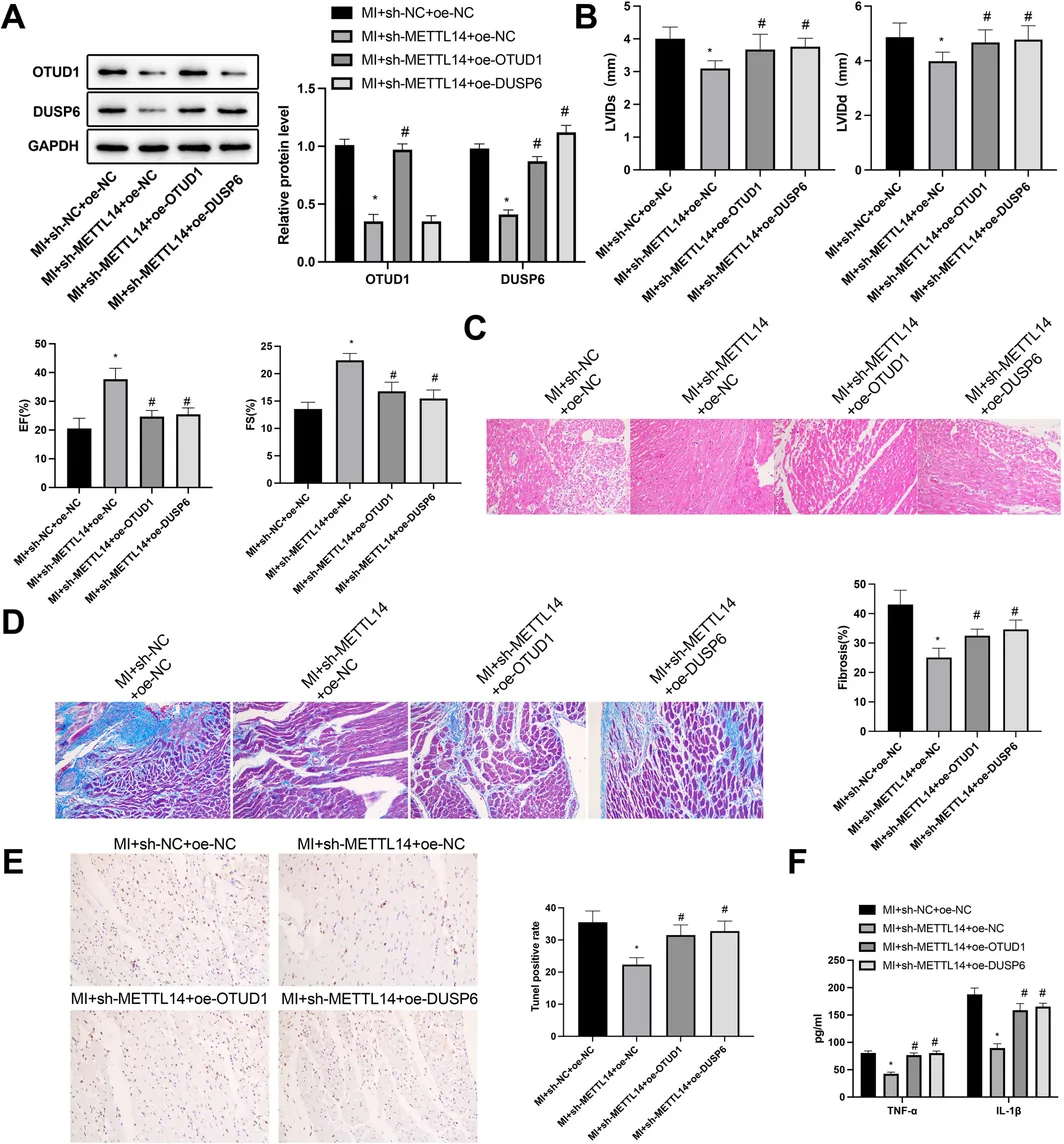
METTL14 knockdown attenuates MI progression in mice via the OTUD1/DUSP6 signaling axis. (A) Western blot analysis of OTUD1 and DUSP6 expression levels. (B) Echocardiographic assessment of cardiac function parameters. (C) HE staining of myocardial tissue to evaluate histopathological changes. (D) Masson's trichrome staining to assess myocardial fibrosis. (E) TUNEL staining to detect cardiomyocyte apoptosis. (F) ELISA analysis of serum TNF‐α and IL‐1β levels. Data are presented as mean ± standard deviation (SD). Comparisons between two groups were performed using unpaired Student's *t*‐test. *p < 0.05 vs. MI + sh‐NC + oe‐NC group; #*p* < 0.05 vs. MI + sh‐METTL14 + oe‐NC group; *n* = 5.

To further elucidate the role of METTL14 in MI, we constructed an MI mouse model with METTL14 knockdown, followed by overexpression of either OTUD1 or DUSP6. Western blot analysis showed that, compared with the MI + sh‐NC + oe‐NC group, the OTUD1 and DUSP6 expression levels were significantly reduced in the MI + sh‐METTL14 + oe‐NC group. In contrast, the OTUD1 and DUSP6 levels were significantly increased in the MI + sh‐METTL14 + oe‐OTUD1 group, while the DUSP6 levels were elevated in the MI + sh‐METTL14 + oe‐DUSP6 group (Figure 4A).

Echocardiographic assessment, HE and Masson staining, TUNEL assays, and ELISA were used to evaluate cardiac function, histopathological changes, fibrosis, apoptosis, and inflammation, respectively. Compared with the MI + sh‐NC + oe‐NC group, the MI + sh‐METTL14 + oe‐NC group exhibited significantly reduced LVIDd and LVIDs, improved EF and FS, alleviated myocardial injury and inflammatory cell infiltration, reduced fibrosis and apoptosis in the infarct border zone, and lower levels of TNF‐α and IL‐1β (Figure 4B–F). However, compared with the MI + sh‐METTL14 + oe‐NC group, both the MI + sh‐METTL14 + oe‐OTUD1 and MI + sh‐METTL14 + oe‐DUSP6 groups showed significantly increased LVIDd and LVIDs, decreased EF and FS, aggravated myocardial pathological damage and inflammation, increased fibrosis and apoptosis, and elevated TNF‐α and IL‐1β levels (Figure 4B–F). These results suggest that METTL14 knockdown attenuates MI‐induced cardiac injury by regulating the OTUD1/DUSP6 signaling axis.

## 4 Discussion

MI remains a leading cause of morbidity and mortality worldwide, characterized by extensive cardiomyocyte death, inflammatory activation, and dysregulated ventricular remodeling. Growing evidence highlights the importance of RNA methylation and protein ubiquitination in heart diseases. However, how m6A RNA methylation interacts with protein ubiquitination in MI remains poorly understood. In this study, we uncovered a novel regulatory axis in which METTL14 modulates MI progression by enhancing OTUD1 m^6^A methylation, thereby influencing DUSP6 ubiquitination and expression.

First, consistent with prior findings showing elevated METTL14 in ischemia–reperfusion (I/R) injury models [29], we observed a significant upregulation of METTL14 in MI mouse hearts. This observation suggests a pathologically relevant induction of METTL14 during myocardial injury, potentially contributing to pathological transcriptional control under stress conditions. Cardiac‐specific silencing of METTL14 has been reported to alleviate acute I/R injury and improve cardiac function during post‐I/R remodeling [30], underscoring the potential role of METTL14 in maintaining cardiac homeostasis. Consistent results were found in the present study, where knockdown of METTL14 mitigated myocardial injury and inflammatory responses, thereby attenuating the progression of MI in mice. METTL14 can catalyze messenger RNA m^6^A modification and is involved in sepsis‐induced myocardial injury via m^6^A‐dependent stabilization of TRPM7 mRNA [31]. In addition, METTL14 was also found to facilitate cardiomyocyte pyroptosis in MI via NLRP3 m6A methylation [7] and m^6^A methylation of pri‐miR‐5099 [8]. Mechanistically, we identified OTUD1 as a direct downstream target whose mRNA stability was enhanced via METTL14‐dependent m^6^A modification. While the role of OTUD1 in cardiovascular pathology is still emerging, recent work has shown that OTUD1 drives pathological cardiac remodeling and HF through the K63‐linked deubiquitination of STAT3 [13]. Beyond its known role as a deubiquitinase, OTUD1 emerges from our study as a downstream target of METTL14, where the m6A‐mediated stabilization of OTUD1 mRNA connects RNA epigenetic regulation with protein ubiquitination in the context of MI. Specifically, METTL14 can upregulate the mRNA stability of OTUD1 through m^6^A modification, suggesting that METTL14 modulates MI progression via m^6^A‐modified OTUD1.

Furthermore, we found that OTUD1 influences the ubiquitination and expression of DUSP6, a dual‐specificity phosphatase that regulates MAPK signaling [17, 32]. Although DUSP6 has been traditionally regarded as a negative regulator of diabetes‐induced cardiac hypertrophy [33], emerging studies reveal its detrimental role in MI, including promoting stress‐induced apoptosis and impairing cardiac repair [18, 19] . Consistent with its role as a negative regulator of the MAPK–ERK pathway, DUSP6 limits cardiomyocyte proliferative capacity, and its suppression can enhance cardiomyocyte proliferation and improve post‐MI cardiac repair [34]. DUSP6 acts as a negative regulator of cardiac regeneration, as its genetic or pharmacological inactivation promotes cardiomyocyte proliferation, enhances coronary angiogenesis, and reduces fibrosis following ventricular resection [35]. A previous study reported that suppression of DUSP6 markedly diminished the cardioprotective effects of TRIM11, underscoring its pivotal role in mediating the beneficial impact of TRIM11 on acute MI progression [20]. Our results demonstrate that METTL14 regulates DUSP6 expression by modulating OTUD1 levels and consequently affecting the ubiquitination of DUSP6. Specifically, METTL14 knockdown markedly reduced METTL14, OTUD1, and DUSP6 expressions, whereas OTUD1 overexpression under METTL14‐silenced conditions significantly restored OTUD1 and DUSP6 expressions. Consistent with previous studies indicating that upregulated DUSP6 expression facilitates MI progression, our work focused on elucidating the regulatory mechanism of the METTL14/OTUD1/DUSP6 axis in MI. Building on these findings, future investigations will aim to clarify how this axis modulates MI progression by influencing specific cellular phenotypes. Importantly, the in vivo knockdown of METTL14 significantly attenuated MI‐induced cardiac dysfunction and remodeling by suppressing the OTUD1/DUSP6 axis. These results underscore the therapeutic potential of targeting METTL14‐dependent m^6^A modification to mitigate post‐infarction responses.

Nonetheless, this study has limitations. It was conducted in murine models, and the relevance to human MI requires further clinical validation. Additionally, the exact m^6^A sites on OTUD1 mRNA, reader proteins involved, and the ubiquitin linkage type on DUSP6 were not characterized. Future directions include mapping m^6^A modification sites, assessing ubiquitin linkage specificity, and evaluating therapeutic interventions targeting this pathway for translational potential.

In summary, this study identified a novel METTL14/OTUD1/DUSP6 regulatory axis in MI. METTL14‐mediated m^6^A modification was found to stabilize OTUD1 transcripts, enabling OTUD1 to modulate DUSP6 ubiquitination and sustain its pathological activity. This insight offers a new mechanistic framework for understanding MI progression and unveils novel targets for intervention.

## Conflicts of Interest

The authors declare no conflicts of interest.

## Data Availability

The data that support the findings of this study are available from the corresponding author upon reasonable request.
